# Medication Adherence and Persistence of Open-Angle Glaucoma Patients in Korea: A Retrospective Study Using National Health Insurance Claims Data

**DOI:** 10.3390/ijerph18084106

**Published:** 2021-04-13

**Authors:** Yunjeong Jang, Donghyun Jee, Donghwan Lee, Nam-Kyong Choi, SeungJin Bae

**Affiliations:** 1 College of Pharmacy, Ewha Womans University, Seoul 03760, Korea; vsjjer@naver.com; 2 St. Vincent’s Hospital, College of Medicine, The Catholic University, Seoul 16247, Korea; doj087@mail.harvard.edu; 3 Department of Statistics, Ewha Womans University, Seoul 03760, Korea; donghwan.lee@ewha.ac.kr; 4 Department of Health Convergence, Ewha Womans University, Seoul 03760, Korea; nchoi@ewha.ac.kr

**Keywords:** open-angle glaucoma, medication adherence, persistence, claim database

## Abstract

This study aimed to analyze medication adherence and persistence among open-angle glaucoma patients in Korea. A retrospective study was conducted using the Korean National Health Insurance (NHI) claims database from 2016 to 2019. Newly diagnosed open-angle glaucoma patients who were prescribed with the intraocular pressure (IOP)-lowering eyedrops were included. Adherence was measured using the medication possession ratio (MPR), and persistence was measured using the duration of therapy during the 24 month follow-up period. During the study period, 14,648 open-angle glaucoma patients were identified, and 3118 (21.3%) and 4481 patients (30.6%) were adherent to and persistent with their glaucoma treatment, respectively. The mean MPR was 48.8%, and the mean duration of therapy was 357.2 days. Logistic regression analysis showed that patients who are older, female, using prostaglandins as the index medication, and visiting secondary or tertiary hospitals were significantly associated with greater rates of adherence (odds ratio (OR) = 1.21, 1.12, 1.27, and 1.73, respectively) and persistence (OR = 1.11, 1.17, 1.16, 1.17, and 1.36, respectively) during the study period. Patients with open-angle glaucoma in Korea had substandard medication adherence and discontinued their treatment. Ophthalmologists should pay more attention to younger, male patients to improve adherence.

## 1. Introduction

Glaucoma is a progressive optic neuropathy disease and one of the major causes of permanent blindness worldwide, affecting more than 70 million people [1,2]. In Korea, the prevalence of glaucoma increased from 0.79% in 2008 to 1.05% in 2013, and it is expected to increase further given the aging population [3]. The goal of glaucoma treatment is to prevent visual field deterioration by lowering the intraocular pressure (IOP). Most glaucoma patients are recommended to be initially treated with topical IOP-lowering eyedrops, including prostaglandin analogs, beta-blockers, carbonic anhydrase inhibitors, and alpha 2-agonists [4]. Among them, prostaglandins and beta-blockers are the drugs most commonly used for initial therapy because they have relatively few adverse effects [5].

For effective glaucoma therapy, patients are required to be adherent (i.e., the extent to which a person behaves according to the prescribed interval and dosing regimen recommended by the provider) and persistent (i.e., to continue with treatment and use prescribed medications for the specific length of time) to lower their IOP and prevent vision loss [6,7]. Poor adherence and persistence are important risk factors related to the progression of glaucoma. Rossi et al. (2010) reported that patients with stable visual fields were more than 75% adherent to their treatment regimen, while patients who recorded less than 45% adherence experienced a worsening of their condition [8]. In addition, patients with asymptomatic open-angle glaucoma are likely to have lower persistence [9]; thus, poor persistence is another risk factor for blindness among glaucoma patients, and it is important to use medication daily without discontinuing treatment [10]. However, few studies have investigated medication adherence and persistence rates of glaucoma patients using nationally representative data, with existing studies limited to assessing only small study populations in certain locations and hospitals [11,12].

There are various factors that may affect medication adherence and persistence rates, including treatment and patient-related factors [13]. Heo et al. (2019) suggested that the use of successful index medications with respect to treatment efficacy, dosing frequency, and adverse events leads to greater medication adherence and persistence [14]. Regarding patient-related factors, Cohen et al. (2014) reported that adherence improves with increasing age [15], while others suggested that age-related conditions including comorbidities and physical or mental difficulties can have negative effects on the medication adherence of older patients [16,17]. In addition, there are inconsistent results regarding sex differences in medication adherence [18,19,20]. Other factors associated with adherence and persistence of glaucoma include environmental factors (e.g., a major life event or unstable lifestyle), provider-related factors (e.g., communication with the doctor), and stage of the disease (e.g., patients with a less advanced disease tend to be less adherent) [4]. Thus, for better treatment outcomes, it is important to assess various factors related to and to identify the characteristics of patients with poor adherence and persistence rates.

This study aimed to analyze medication adherence and persistence among patients with open-angle glaucoma who started treatment using IOP-lowering glaucoma medications and to identify factors linked to poor adherence and persistence using the Korean National Health Insurance (NHI) claims database.

## 2. Materials and Methods

### 2.1. Data Source

We conducted a population-based, retrospective study from 1 February 2016, to 31 July 2019, using the Korean National Health Insurance (NHI) claims database, which covers almost 98% of the total Korean population (approximately 50 million people) [21]. The Korean NHI claims database provides key information such as patients’ demographic characteristics, health insurance types, diagnosis codes, treatment costs, and prescription details [21]. To protect patients’ personal information, all identification numbers of patients are encrypted [22]. The study was approved by the institutional review board of Ewha Womans University (protocol code ewha-201912-0021-01).

### 2.2. Study Population

**Figure 1:**
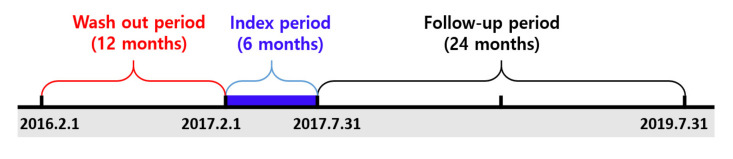
Study design.

The study population consisted of patients newly diagnosed with open-angle glaucoma and who were older than 20 years during the index period (from 1 February 2017, to 31 July 2017). To identify newly diagnosed patients, we included those diagnosed with open-angle glaucoma (International Classification of Disease-10th Revision (ICD-10) code H401) based on their primary or secondary diagnosis with prescription of prostaglandins or beta-blockers. The first date of open-angle glaucoma diagnosis was set as the index date. Patients were required to have at least two claims for glaucoma medications during follow-up. We excluded patients with an open-angle glaucoma diagnosis in the 12 months before the index date in order to exclude patients who had been diagnosed previously. Based on a prior study [23], patients who were primarily diagnosed with diabetic retinopathy, retinitis, and disorders of the vitreous body were excluded from our study population. The follow-up period for measuring medication adherence and persistence of glaucoma patients was defined as 24 months after the index date [14]. The study design is shown in Figure 1.

### 2.3. Outcomes

We estimated the overall adherence of glaucoma medication using the medication possession ratio (MPR), which is the percentage of total days for which the patient has their medication during the observation period [24,25]. We calculated the MPR as the number of total days’ supply during which the patient possesses the medication available according to the prescription divided by the total observation period (730 days). Since estimating the supply of eyedrops using claims data is known to be an inaccurate technique, we additionally used the drop-count method, which determines the actual volume and drops of drug solution dispensed from each bottle [26]. The number of drops per bottle was determined from previous studies [7,27,28,29]. Patients with an MPR of at least 80% were defined as adherent patients; otherwise, patients were defined as nonadherent [6]. Persistence was identified by measuring the duration of therapy, which is the time from the date of the first prescription of the index medication to the discontinuation of therapy. Patients were considered to have discontinued therapy when there was at least a 90 day gap between consecutive prescriptions of glaucoma medications [14,30]. Patients were considered as persistent when there was no discontinuation of therapy during the 24 month follow-up period. We also conducted a sensitivity analysis for adherence using MPR of at least 70% or at least 50% as cutoff values and for persistence using prescription gaps of 60 days and 120 days, respectively.

### 2.4. Statistical Analysis

Demographic characteristics, MPR, and the duration of therapy were compared among patient groups using *t*-tests for continuous variables and chi-squared tests for categorical variables. Given that adherence and persistence have binary outcomes (yes/no), we performed logistic regression analysis to identify associations between independent and outcome variables. Additionally, the GLM was used to assess medication adherence and persistence as continuous outcomes. Independent variables were a type of index medication (beta-blockers = 0, prostaglandins = 1), age (<60 = 0, ≥60 = 1), sex (male = 0, female = 1), Charlson comorbidity index (CCI) score (score 0 = 0, score ≥1 = 1), medical institution (primary care = 0, secondary or tertiary care = 1), and comorbidities of hypertension, diabetes, or stroke (No = 0, Yes = 1). All analyses were conducted using SAS version 9.4 (SAS Institute Inc., Cary, NC, USA). The significance level was set at *p* < 0.05.

## 3. Results

### 3.1. Adherence, Persistence, and Demographic Characteristics

During the study period, a total of 14,648 patients newly diagnosed with open-angle glaucoma and treated initially with prostaglandins or beta-blockers as monotherapy were included. The MPR, duration of therapy, and demographic characteristics of the study population are shown in Table 1. Most patients were 60 years or older (68.5%), female (52.0%), used prostaglandin as index medication (86.0%), had CCI scores greater than zero (55.2%), and were treated at a primary hospital (83.2%). In terms of the comorbidity, 45.5%, 24.3%, and 5.1% of those patients had hypertension, diabetes, and stroke, respectively. Among the total study population, the mean MPR was 48.8%, and the mean duration of therapy was 357.2 days. There was a significant difference in MPR and duration of therapy between adherent and nonadherent and persistent and nonpersistent patients, respectively. Of the 14,648 patients, 3118 (21.3%) were adherent (MPR ≥ 80%), and 4481 (30.6%) were persistent with their glaucoma treatment during 24 months of follow-up. In addition, 2923 patients (19.9%) were both adherent and persistent with their treatment. With regard to sensitivity analysis, the proportions of adherent patients were 28.9% and 45.9% when the cutoff was defined as at least 70% and at least 50%, respectively. In addition, the mean durations of therapy were 306.3 days and 389.4 days when the prescription gap was defined as 60 days and 120 days, respectively.

The proportion of adherent patients who were older than 60 years of age (22.2%) was significantly higher than that of patients who were younger than 60 years (19.3%, *p* < 0.0001). The proportion of adherent patients who initiated therapy with prostaglandins (21.9%) was also significantly higher than that of patients who initiated therapy with beta-blockers (17.7%, *p* < 0.0001). Regarding sex, the percentage of adherent female patients (22.1%) was significantly higher than that of male patients (20.4%, *p* = 0.0141). More than 80% of total patients were treated in a primary hospital, but the proportion of adherent patients who were treated in secondary or tertiary hospitals (29.5%) was significantly higher than that of patients who were treated in primary hospitals (19.6%, *p* < 0.0001). These demographic differences showed the same trend in relation to persistence status: the proportion of persistent patients was significantly higher among those who were older than 60 years (31.4 vs. 28.9%, *p* = 0.0031), who initiated therapy with prostaglandins (31.0 vs. 27.8%, *p* = 0.0033), female patients (32.1 vs. 28.9%, *p* < 0.0001), and those who were treated in secondary or tertiary hospitals (36.0 vs. 29.5%, *p* < 0.0001). Meanwhile, though the percentage of adherent patients with CCI scores of more than zero points (22.0%) was significantly higher than that of patients with CCI scores being zero (20.4%, *p* = 0.0188), there was no significant difference between these CCI score groups regarding persistent patients (*p* = 0.6633).

### 3.2. Logistic Regression Analysis for Adherence and Persistence

Table 2 shows the results of univariate and multivariate logistic regression analyses for adherence and persistence. In the univariate analysis, all variables except CCI score and comorbidities (hypertension, diabetes, and stroke) were significantly associated with both adherence and persistence. Higher CCI scores were significantly associated with greater medication adherence (OR: 1.10, *p* = 0.0315) but not with persistence (*p* = 0.4103). Multivariate analysis demonstrated that patients who were older than 60 years, those who initiated therapy with prostaglandins, female patients, and those who were treated in secondary or tertiary hospitals had significantly higher odds of medication adherence (OR: 1.21, *p* < 0.001; OR: 1.27, *p* < 0.001; OR: 1.12, *p* < 0.01; OR: 1.73, *p* < 0.001, respectively) and persistence (OR: 1.11, *p* < 0.01; OR: 1.16, *p* < 0.01; OR: 1.17, *p* < 0.001; OR: 1.36, *p* < 0.001, respectively). Additionally, GLM was used to assess adherence and persistence as continuous variables. Multivariate GLM showed that patients who were older than 60 years, who initiated therapy with prostaglandins, female patients, and those who were treated in secondary or tertiary hospitals had significantly higher MPR (β = 0.049, *p* < 0.001; β = 0.110, *p* < 0.001; β = 0.056, *p* < 0.001; β = 0.151, *p* < 0.001, respectively) and longer duration of therapy (β = 0.036, *p* < 0.05; β = 0.074, *p* < 0.001; β = 0.073, *p* < 0.001; β = 0.162, *p* < 0.001, respectively). The results are shown in Table A1.

Descriptive analysis was conducted to compare mean MPR and duration of therapy of the study population. The results showed that mean MPR and duration of therapy were significantly higher and longer among those older than 60 years (49.7 vs. 46.8%, *p* < 0.0001; 362 vs. 346 days, *p* = 0.0009), who initiated therapy with prostaglandins (49.5 vs. 44.3%, *p* < 0.0001; 361 vs. 335 days, *p* < 0.0001), female patients (50.0 vs. 47.4%, *p* < 0.0001; 369 vs. 334 days, *p* < 0.0001), and those who were treated in secondary or tertiary hospitals (55.1 vs. 47.5%, *p* < 0.0001; 410 vs. 347 days, *p* < 0.0001). The results are shown in Table 3.

### 3.3. Adherence, Persistence, and Characteristics by Index Medication

The MPR, duration of therapy, and demographic characteristics of the study population by index medication are shown in Table 4. The mean MPR among patients who used prostaglandins and beta-blockers as the index medication were 49.5% and 44.3%, respectively (*p* < 0.0001). The mean duration of therapy also showed significant difference between prostaglandins and beta-blockers users (360.8 vs. 334.9 days, *p* < 0.0001). Regarding demographic characteristics, the proportion of prostaglandin users as the index medication was significantly higher among males (88.0 vs. 84.1%, *p* < 0.0001), patients with higher number of comorbidities (86.5 vs. 85.3%, *p* = 0.0433), and patients who were treated in secondary or tertiary hospitals (90.1 vs. 85.1%, *p* < 0.0001).

## 4. Discussion

### 4.1. Adherence and Persistence in Korea

Our study assessed medication adherence and persistence among open-angle glaucoma patients who received IOP lowering eyedrops using Korean NHI claims data. MPR, which is one of the most common methods for estimating adherence using retrospective data, was used to measure the overall medication adherence [31]. The mean MPR was 48.8%, and just 21.3% of the patients were adherent (MPR ≥ 80%) to their glaucoma medications during the study period. The mean duration of therapy among total patients was 357.2 days out of 730 days, and 30.6% of study participants were persistent with their treatment. This suggests that most open-angle glaucoma patients in Korea discontinue their therapies within 24 months of follow-up; thus, efforts to improve medication adherence and persistence are necessary. Furthermore, several studies found that normal-tension glaucoma, which is open-angle glaucoma with low IOP, was the most common type in Korean populations [32,33,34]. Since normal-tension glaucoma patients typically have low IOP that are consistently at or below 21 mmHg [35], they might not be aware of the severity of glaucoma and importance of adherence and persistence. In adddition, more intensive treatments and observations are important because IOP alone is not reliable to determine the severity and progression of glaucoma [35], which emphasizes the need for adherence and persistence of glaucoma patients in Korea.

Our results are somewhat lower than those values reported in the previous studies [26,28,36]. Sheer et al. (2016) showed that the mean adherence rate of patients aged 65–89 years in the United States was 72.0% [26], which was much higher than what we found in our study. However, the previous study estimated adherence for a 12 month follow-up period among patients older than 65 [26], whereas we investigated patients older than 20 years for 24 months of follow-up; thus, a shorter follow-up period and including only older patients might have led to the higher adherence rate in the former investigation. In addition, compared with the results of Sheer et al., we reported a 61.2% mean adherence rate among patients older than 65 years during 12 months of follow-up; however, the difference in results might have been influenced by the definition of the study population since the previous study included both new and continuing users of glaucoma medications, whereas we only assessed newly diagnosed patients. Likewise, Friedman et al. (2007) showed that the mean MPR of 13,977 patients using drop count method was 64% [28], which was higher than our result. The sample size, follow-up period, and methods were similar to those of our study, but the wash-out period was only 6 months and patients were 40 years of age or older, which might have affected the higher MPR. There are few studies to date that have investigated medication adherence and persistence among glaucoma patients in Korea, and no study has used claims data. One Korean study conducted in 15 nationwide medical institutions in Korea reported that the mean adherence rate of glaucoma patients was 90.6% [36], but this result was based on the collection of self-reported questionnaires from 1050 patients older than 20 years who had already been treated for at least one month before the study period. The medication adherence rate was measured by calculating the proportion of the number of times patients missed administering eyedrops during the study period using patient questionnaires, which might impact the rate of adherence, whereas our study assessed the MPR using NHI claims data. Furthermore, patients’ adherence was assessed for only seven days, which was much shorter than our study period. Further long-term studies targeting the entire Korean population are needed.

### 4.2. Factors Associated with Adherence and Persistence

There are known patient-related risk factors for poor adherence, including patients’ knowledge and attitude. Previous studies have reported that poor knowledge of diseases, a lack of understanding of the goal of glaucoma treatment, and less-aggressive attitudes toward medical information are associated with decreased medication adherence rates [37,38,39]. Our research suggests that older patients are more likely to be adherent to and persistent with their medication regimen than younger patients. Open-angle glaucoma is asymptomatic in the early stages, and vision loss progresses slowly, which may be a reason for why younger patients are not fully aware of the importance of taking their medication, resulting in their lower adherence and persistence rates [16,40]. We also found that female patients were more likely to be adherent to and persistent with their medication than male patients, possibly because women may visit physicians to a greater extent than men [41]. Additionally, female patients are more likely to have higher levels of health literacy than male patients, which means that female patients tend to understand medical forms, directions on taking medication, and information offered by healthcare providers better than male patients [42]. Therefore, it is likely that male and younger patients in Korea have poorer perceptions of glaucoma treatment and adherence. It is necessary to pay special attention and to provide aggressive counseling to them to improve their medication adherence and persistence. The use of educational programs can also help to improve adherence and persistence by motivating them to actively participate in their treatment [43].

In addition, our study found that patients who were treated in secondary or tertiary hospitals were more likely to be adherent and persistent. Mathews et al. (2018) reported that high adherence rates are associated with larger hospitals and academic centers, which may provide overall higher quality of services to patients [44]. Therefore, it is expected that patients’ adherence and persistence can be improved by providing high-quality medical services to increase the patients’ satisfaction and by providing sufficient education and information to improve patients’ awareness of the need for and motivation to pursue medication adherence.

Patients who initiated therapy with prostaglandins were more likely to be adherent and persistent than those who initiated with beta-blockers, which may be explained by the greater efficacy of lowering IOP, fewer systemic side effects, and simpler dosing strategy (once-a-day dose) inherent with prostaglandins [45]. Nordstrom et al. (2005) reported similar results stipulating that prostaglandin use was associated with better medication adherence [20]. Healthcare providers might help patients to improve adherence and persistence by considering effective and simplified regimens, including prostaglandins. We additionally compared the patients who used prostaglandins and beta-blockers as the index medication to identify the characteristics of prostaglandin users. Males, patients with higher CCI scores, and those who were treated in secondary or tertiary hospitals are more likely to use prostaglandins as the index medication than beta-blockers. The lower proportion of female patients who used prostaglandins as the index medication may be related to common side effects of prostaglandins, such as hyperemia, eyelid pigmentation, and eyelash change in color or number. [46,47] These aesthetically unfavorable side effects could be a reason of lower use of prostaglandins among female patients. In addition, patients with higher CCI scores might take multiple medications, and they might be reluctant to use beta-blockers since they are associated with systemic side effects and complex dosing strategy (twice-a-days dose) [5]. Lower proportion of prostaglandin users who were treated in primary hospitals could be interpreted from characteristics of primary hospitals. Local primary hospitals tend to prefer generic or cheaper medications according to the patients’ preference [48,49].

In our study, patients with higher CCI scores were more likely to be adherent but not persistent. In the case of patients with severe comorbidities, these individuals might receive long-term prescriptions of glaucoma medications due to the difficulty in attending frequent outpatient visits. They might also exhibit higher MPR but lower persistence because a 90 day gap between consecutive prescriptions was used to define persistence in our study. Thus, there is a need to improve persistence by providing enhanced monitoring systems to prevent poor medication persistence among patients with severe comorbidities.

### 4.3. Limitations and Strengths

Our study has some limitations. First, since there was no clinical information available in the claims database, we cannot explain how the results of our study were related to clinical characteristics and outcomes, including disease severity and IOP, and it is not possible to identify the reason for discontinuation of treatment. Secondly, Korea Health Insurance Review and Assessment (HIRA) provides NHI claims data with the limitation of up to 100 million prescriptions, and the 24 month follow up period was chosen to include as many patients as possible, which might have underestimated the MPR. Finally, MPR indicates only the possession of medication, and we cannot determine whether the medication was actually ingested by the patients. Since the patients were assumed to have used all obtained medications, there is a possibility of the overestimation of actual adherence [24].

Despite these limitations, to our knowledge, our study is the first population-based study in Korea to estimate medication adherence and persistence of patients with open-angle glaucoma using national claims data. The Korean NHI claims database includes information of almost 50 million people, covering 98% of the total population in Korea. This representativeness of claims data is a great advantage that is not feasible in randomized controlled trials, which are limited to including small sample size and limited conditions [21]. Additionally, using claims data to measure adherence could be more objective, whereas measuring adherence from self-report or survey information is highly dependent on patients’ recall [25].

Our study showed the medication adherence and persistence of open-angle glaucoma patients using population-based, real-world data. The MPR of Korean open-angle glaucoma patients seems to be low, and many patients stopped adhering to their therapy plan during the study period. For better adherence and persistence, ophthalmologists should pay more attention to male or younger patients and strive to improve their adherence by providing more intensive education, monitoring, and high-quality services and by considering adopting more simplified dosing strategies, including prostaglandins. Although treatment options should be considered based on the clinical characteristics of individual patients, the results of our study support ophthalmologists when they make decisions among many alternatives.

## 5. Conclusions

Many patients with open-angle glaucoma in Korea exhibited low medication adherence and discontinued their treatment during the study period. Ophthalmologists should pay attention in particular to male or younger patients with poor adherence and persistence rates and try to improve their adherence and persistence.

## Figures and Tables

**Table 1:** Demographic characteristics, adherence, and persistence of patients with open-angle glaucoma.

Variable	Total (n = 14,648)	Adherence Status ^1^			Persistence Status ^2^		
		Adherent (n = 3118)	Nonadherent (n = 11,530)	*p*-Value ^3^	Persistent (n = 4481)	Nonpersistent (n = 10,167)	*p*-Value ^3^
MPR (%), mean ± SD ^4^	48.8 ± 30.5	93.4 ± 6.6	36.7 ± 22.1	<0.0001	84.0 ± 16.2	33.3 ± 21.0	<0.0001
Duration of therapy (days), mean ± SD ^5^	357.2 ± 276.2	706.1 ± 95.2	262.8 ± 229.4	<0.0001	722.3 ± 17.6	196.3 ± 158.5	<0.0001
**Age, years**							
20–59	4621 (31.5)	890 (19.3)	3731 (80.7)	<0.0001	1337 (28.9)	3284 (71.1)	0.0031
≥60	10,027 (68.5)	2228 (22.2)	7799 (77.8)		3144 (31.4)	6883 (68.6)	
**Type of index medication**							
Beta-blockers	2056 (14.0)	363 (17.7)	1693 (82.3)	<0.0001	572 (27.8)	1484 (72.2)	0.0033
Prostaglandin analogs	12,592 (86.0)	2755 (21.9)	9837 (78.1)		3909 (31.0)	8683 (69.0)	
**Sex**							
Male	7027 (48.0)	1435 (20.4)	5592 (79.6)	0.0141	2034 (28.9)	4993 (71.1)	<0.0001
Female	7621 (52.0)	1683 (22.1)	5938 (77.9)		2447 (32.1)	5174 (67.9)	
**CCI**							
0	6567 (44.8)	1340 (20.4)	5227 (79.6)	0.0188	2021 (30.8)	4546 (69.2)	0.6633
≥1	8081 (55.2)	1778 (22.0)	6303 (78.0)		2460 (30.4)	5621 (69.6)	
**Medical institution**							
Primary care	12,181 (83.2)	2391 (19.6)	9790 (80.4)	<0.0001	3592 (29.5)	8589 (70.5)	<0.0001
Secondary or tertiary care	2467 (16.8)	727 (29.5)	1740 (70.5)		889 (36.0)	1578 (64.0)	
**Hypertension**							
No	7986 (54.5)	1664 (20.8)	6322 (79.2)	0.1454	2419 (30.3)	5567 (69.7)	0.3872
Yes	6662 (45.5)	1454 (21.8)	5208 (78.2)		2062 (30.9)	4600 (69.1)	
**Diabetes**							
No	11,082 (75.7)	2343 (21.1)	8739 (78.9)	0.4536	3418 (23.3)	7664 (69.2)	0.2440
Yes	3566 (24.3)	775 (21.7)	2791 (78.3)		1063 (29.8)	2503 (70.2)	
**Stroke**							
No	13,906 (94.9)	2954 (21.2)	10,952 (78.8)	0.5772	4269 (30.7)	9637 (69.3)	0.2204
Yes	742 (5.1)	164 (22.1)	578 (77.9)		212 (28.6)	530 (71.4)	

^1^ Patients were considered adherent when MPR ≥ 80%. ^2^ Patients were considered persistent when there was less than a 90 day gap between consecutive prescriptions. ^3^ Results were significant at *p* < 0.05. A *t*-test for continuous variables and chi-square test for categorical variables were used. ^4^ MPR (medication possession ratio) = Sum of days’ supply during the observation period/total observation period. ^5^ Duration of therapy is the time from the date of the first prescription of the index medication to the discontinuation of therapy. SD, standard deviation; CCI, Charlson comorbidity index.

**Table 2:** Logistic regression analysis for medication adherence and persistence.

Variable	Adherence Status ^1^		Persistence Status ^2^	
	Univariate	Multivariate	Univariate	Univariate
**Age, years**				
20–59	1 (reference)	1 (reference)	1 (reference)	1 (reference)
≥60	1.20 (1.10, 1.31) ^‡^	1.21 (1.10, 1.32) ^‡^	1.12 (1.04, 1.21) ^†^	1.11 (1.03, 1.20) ^†^
**Type of index medication**				
Beta-blockers	1 (reference)	1 (reference)	1 (reference)	1 (reference)
Prostaglandins	1.31 (1.16, 1.47) ^‡^	1.27 (1.13, 1.44) ^‡^	1.17 (1.05, 1.30) ^†^	1.16 (1.05, 1.29) ^†^
**Sex**				
Male	1 (reference)	1 (reference)	1 (reference)	1 (reference)
Female	1.10 (1.02, 1.20) *	1.12 (1.04, 1.22) ^†^	1.16 (1.08, 1.25) ^‡^	1.17 (1.09, 1.26) ^‡^
**CCI**				
0	1 (reference)	1 (reference)	1 (reference)	
≥1	1.10 (1.02, 1.19) *	1.00 (0.92, 1.10)	0.98 (0.92, 1.06)	
**Medical institution**				
Primary care	1 (reference)	1 (reference)	1 (reference)	1 (reference)
Secondary or tertiary care	1.71 (1.55, 1.89) ^‡^	1.73 (1.57, 1.91) ^‡^	1.35 (1.23, 1.48) ^‡^	1.36 (1.24, 1.49) ^‡^
**Hypertension**				
No	1 (reference)		1 (reference)	
Yes	1.06 (0.98, 1.15)		1.03 (0.96, 1.11)	
**Diabetes**				
No	1 (reference)		1 (reference)	
Yes	1.04 (0.95, 1.14)		0.95 (0.88, 1.03)	
**Stroke**				
No	1 (reference)		1 (reference)	
Yes	1.05 (0.88, 1.26)		0.903 (0.77, 1.06)	

^1^ Adherence was defined as an event and values are given as odds ratios (95% confidence intervals). ^2^ Persistence was defined as an event and values are given as odds ratios (95% confidence intervals). * *p* value < 0.05. ^†^ *p* value < 0.01. ^‡^ *p* value < 0.001. CCI, Charlson comorbidity index.

**Table 3:** Mean MPR (medication possession ratio) and duration of therapy.

Variable	MPR (%), Mean ± SD ^1^	*p*-Value ^2^	Duration of Therapy (Days), Mean ± SD ^3^	*p*-Value ^2^
**Total patients (n = 14,648)**	48.8 ± 30.5	-	357.2 ± 276.2	-
**Age, years**				
20–59	46.8 ± 30.4	<0.0001	346.0 ± 274.3	0.0009
≥60	49.7 ± 30.6		362.3 ± 276.9	
**Type of index medication**				
Beta-blockers	44.3 ± 30.5	<0.0001	334.9 ± 275.9	<0.0001
Prostaglandin analogs	49.5 ± 30.5		360.8 ± 276.1	
**Sex**				
Male	47.4 ± 30.6	<0.0001	334.2 ± 274.6	<0.0001
Female	50.0 ± 30.5		369.2 ± 277.1	
**CCI**				
0	48.4 ± 30.3	0.2419	357.7 ± 276.7	0.8481
≥1	49.0 ± 30.7		256.8 ± 275.8	
**Medical institution**				
Primary care	47.5 ± 30.2	<0.0001	347.1 ± 276.7	<0.0001
Secondary or tertiary care	55.1 ± 31.3		409.8 ± 268.1	
**Hypertension**				
No	48.2 ± 47.5	0.0160	353.8 ± 276.1	0.0996
Yes	49.4 ± 30.4		361.3 ± 276.3	
**Diabetes**				
No	48.8 ± 30.5	0.9215	357.9 ± 276.5	0.5604
Yes	48.8 ± 30.6		354.9 ± 275.2	
**Stroke**				
No	48.8 ± 30.5	0.8218	357.5 ± 276.5	0.5742
Yes	49.0 ± 30.9		351.6 ± 269.9	

^1^ MPR (medication possession ratio) = Sum of days’ supply during the observation period/total observation period. ^2^ Results were significant at *p* < 0.05 according to a *t*-test. ^3^ Duration of therapy is the time from the date of the first prescription of the index medication to the discontinuation of therapy. SD = standard deviation, CCI = Charlson comorbidity index.

**Table 4:** Demographic characteristics of patients with open-angle glaucoma by index medication.

Variable	Total (n = 14,648)	Type of Index Medication		
		Prostaglandins (n = 12,592)	Beta-Blockers (n = 2056)	*p*-Value ^1^
**MPR (%), mean ± SD ^2^**	48.8 ± 30.5	49.5 ± 30.5	44.3 ± 30.5	<0.0001
**Adherence status, n (%) ^3^**				
Adherent	3118 (21.3)	2755 (21.9)	363 (17.7)	<0.0001
Nonadherent	11,530 (78.7)	9837 (78.1)	1693 (82.3)	
**Duration of therapy (days), mean ± SD ^4^**	357.2 ± 276.2	360.8 ± 276.1	334.9 ± 275.9	<0.0001
**Persistence status, n (%) ^5^**				
Persistent	4481 (30.6)	3909 (31.0)	572 (27.8)	0.0033
Nonpersistent	10,167 (69.4)	8683 (69.0)	1484 (72.2)	
**Age, years**				
20–59	4621 (31.5)	3935 (85.2)	686 (14.8)	0.0556
≥60	10,027 (68.5)	8657 (86.3)	1370 (13.7)	
**Sex**				
Male	7027 (48.0)	6186 (88.0)	841 (12.0)	<0.0001
Female	7621 (52.0)	6406 (84.1)	1215 (15.9)	
**CCI**				
0	6567 (44.8)	5603 (85.3)	964 (14.7)	0.0433
≥1	8081 (55.2)	6989 (86.5)	1092 (13.5)	
**Medical institution**				
Primary care	12,181 (83.2)	10,370 (85.1)	1811 (14.9)	<0.0001
Secondary or tertiary care	2467 (16.8)	2222 (90.1)	245 (9.9)	
**Hypertension**				
No	7986 (54.5)	6875 (86.1)	1111 (13.9)	0.6356
Yes	6662 (45.5)	5717 (85.8)	945 (14.2)	
**Diabetes**				
No	11,082 (75.7)	9511 (85.8)	1571 (14.2)	0.3895
Yes	3566 (24.3)	3081 (86.4)	485 (13.6)	
**Stroke**				
No	13,906 (94.9)	11,941 (85.9)	1965 (14.1)	0.1538
Yes	742 (5.1)	651 (87.7)	91 (12.3)	

^1^ Results were significant at *p* < 0.05. A *t*-test for continuous variables and chi-square test for categorical variables were used. ^2^ MPR (medication possession ratio) = sum of days’ supply during the observation period/total observation period. ^3^ Patients were considered adherent when MPR ≥ 80%. ^4^ Duration of therapy is the time from the date of the first prescription of the index medication to discontinuation of therapy. ^5^ Patients were considered persistent when there was less than a 90 day gap between consecutive prescriptions. SD = standard deviation, CCI = Charlson comorbidity index.

## Data Availability

Data sharing is not applicable to this article.

## Appendix A

**Table A1:** Generalized linear model for medication adherence and persistence.

Variable	Medication Possession Ratio (MPR) ^1,2^		Duration of Therapy ^1,2^	
	Univariate	Multivariate	Univariate	Multivariate
**Age, years**				
20–59	-	-	-	-
≥60	0.059 (0.034, 0.084) ^‡^	0.049 (0.023, 0.076) ^‡^	0.046 (0.015, 0.078) ^†^	0.036 (0.005, 0.067) *
**Type of index medication**				
Beta-blockers	-	-	-	-
Prostaglandins	0.111 (0.078, 0.144) ^‡^	0.110 (0.077, 0143) ^‡^	0.074 (0.034, 0.115) ^‡^	0.074 (0.033, 0.115) ^‡^
**Sex**				
Male	-	-	-	-
Female	0.054 (0.030, 0.077) ^‡^	0.056 (0.032, 0.079) ^‡^	0.070 (0.042, 0.098) ^‡^	0.073 (0.045, 0.102) ^‡^
**CCI**				
0	-		-	
≥1	0.012 (−0.011, 0.035)		−0.003 (−0.031, 0.026)	
**Medical institution**				
Primary care	-	-	-	-
Secondary or tertiary care	0.148 (0.117, 0.179) ^‡^	0.151 (0.120, 0.182) ^‡^	0.159 (0.121, 0.197) ^‡^	0.162 (0.124, 0.200) ^‡^
**Hypertension**				
No	-	-	-	
Yes	0.025 (0.002, 0.048) *	0.008 (−0.017, 0.032)	0.021 (−0.008, 0.050)	
**Diabetes**				
No	-		-	
Yes	−0.001 (−0.028, 0.026)		−0.009 (−0.042, 0.025)	
**Stroke**				
No	-		-	
Yes	0.005 (−0.047, 0.058)		−0.017 (−0.081, 0.048)	

^1^ Generalized linear model with gamma distribution and the log link function were used for medication adherence and persistence. ^2^ Values are given as β (95% confidence intervals). * *p* value < 0.05. † *p* value < 0.01. ^‡^ *p* value < 0.001. CCI = Charlson comorbidity index.
